# Editorial: Neuromorphic computing: from emerging materials and devices to algorithms and implementation of neural networks inspired by brain neural mechanism

**DOI:** 10.3389/fncom.2024.1443758

**Published:** 2024-07-19

**Authors:** Guohe Zhang

**Affiliations:** School of Microelectronics, Key Lab of Micro-Nano Electronics and System Integration of Xi'an City, Xi'an Jiaotong University, Xi'an, China

**Keywords:** spiking neural networks, reservoir computing (RC), photonic hardware, neuroAIx-Framework, computational neuroscience

One of the key challenges in computational neuroscience is the need for powerful simulation platforms to study complex neural network models. Traditional simulators often struggle to keep up with the complexity and scale of these models, leading to slow simulation times and limited insights. To address this challenge, Kauth et al. introduced the neuroAIx-Framework, a flexible and scalable approach to explore future neuroscience simulation systems. This framework leverages empirical modeling tools, virtual prototypes, and FPGA clusters to achieve significant acceleration, even for large-scale models. The FPGA cluster developed within this framework achieves a remarkable 20 × acceleration compared to biological real-time, setting a new benchmark in the field. This breakthrough opens doors for researchers to study complex neural processes and explore new hypotheses at a scale previously unimaginable.

Spiking Neural Networks (SNNs) have gained attention for their potential to achieve low-power and low-latency AI computations. Traditional neural network architectures, such as LSTM and CNN, are energy-intensive and not suitable for resource-constrained devices. SNNs, on the other hand, exhibit spike-based computations that closely resemble biological neural processing and offer significant energy savings. Gaurav et al. presented two novel SNN architectures for univariate Time Series Classification (TSC), inspired by Reservoir Computing and Legendre Memory Units. The proposed models, SLRC, and LSNN, demonstrate promising results on various TSC datasets and showcase the potential of SNNs for energy-efficient AI applications. The LSNN model, in particular, achieves state-of-the-art spiking results while significantly reducing the number of neurons compared to traditional LSM-based models. This demonstrates the potential of SNNs for developing low-power AI systems that can operate on battery-powered devices, such as IoT sensors and wearable devices.

Photonic hardware-based neural networks offer promising avenues for fast and energy-efficient image recognition tasks. Traditional electronic hardware, such as GPUs and FPGAs, are limited by the speed of electrical signals and cannot match the parallelism and speed of photonic systems. Masominia et al. explored the use of ultrafast photonic spiking neurons for digit classification, demonstrating the potential of event-based, spike-time, and rank-order based algorithms. These techniques leverage the parallelism of photonic hardware and minimize the number of spiking nodes required for a given task, potentially revolutionizing image recognition systems. This opens doors for developing ultrafast and energy-efficient vision systems for applications such as autonomous vehicles, medical imaging, and surveillance.

Reservoir Computing (RC) is a powerful paradigm for machine learning, offering efficient and effective solutions for various tasks. Calvet, Rouat et al. and Calvet, Reulet et al. investigated the relationship between connectivity, dynamics, and performance in Random Boolean Networks (RBNs), a popular model for RC. The studies revealed that the excitatory-inhibitory balance plays a crucial role in driving dynamics and performance, overriding attractor dynamics. The findings provide valuable insights for designing optimal RBN reservoirs and simplifying the reservoir design process. This understanding can lead to the development of more efficient and effective RC models that can be used for a wide range of applications, such as speech recognition, natural language processing, and signal processing.

The articles discussed above showcase the diverse and exciting research happening in computational neuroscience. The future of the field holds several promising directions:

1. Developing More Complex SNN Architectures: Exploring more complex SNN architectures that can leverage the inherent temporal dynamics of spiking neurons for various AI tasks, such as object recognition, speech understanding, and decision-making.
2. Combining SNNs and Reservoir Computing: Exploring the potential of combining SNNs and Reservoir Computing to achieve even higher performance and efficiency, and enabling new applications in AI and neuroscience.
3. Developing Brain-Inspired Computing Platforms: Building brain-inspired computing platforms that can efficiently simulate complex neural network models and enable new discoveries in neuroscience, leading to the development of more efficient and intelligent AI systems.
4. Expanding the Scope of Applications: Applying computational neuroscience techniques to a wider range of domains, including healthcare, robotics, and brain-computer interfaces, to develop innovative solutions and improve human lives.

Computational Neuroscience is at the forefront of AI and neuroscience research, offering exciting opportunities to revolutionize our understanding of the brain and develop new AI applications. The recent advancements in simulation platforms, spiking neural networks, photonic hardware, and reservoir computing showcase the potential of this field to shape the future of AI and neuroscience. I am confident that the collaborative efforts of researchers across the globe will continue to drive innovation and breakthroughs in computational neuroscience, leading to a brighter and more connected future.

## Author contributions

GZ: Writing – original draft, Writing – review & editing.

